# Ocular Surface Findings in Patients with Surgery-Indicated Chronic Rhinosinusitis with Nasal Polyps: A Retrospective Study

**DOI:** 10.3390/diagnostics16203352

**Published:** 2026-10-15

**Authors:** Efnan Abdioğlu Fazlı, Büşra Köse, Ömer K. Kurutaş, Osman Bahadır

**Affiliations:** 1 Department of Otorhinolaryngology, Faculty of Medicine, Karadeniz Technical University, Trabzon 61080, Turkey; osman_bahadir@yahoo.com; 2 Department of Ophthalmology, Faculty of Medicine, Karadeniz Technical University, Trabzon 61080, Turkey; busrakose-ktu@hotmail.com (B.K.); omerkoksalkurutas@hotmail.com (Ö.K.K.)

**Keywords:** chronic rhinosinusitis, nasal polyps, dry eye disease, ocular surface, Lund–Mackay score

## Abstract

**Background/Objectives:** Inflammation plays a central role in the pathophysiology of both chronic rhinosinusitis (CRS) and dry eye disease (DED), suggesting a possible link between these two conditions. This study aimed to investigate the relationship between surgery-indicated chronic rhinosinusitis with nasal polyps (CRSwNP) and ocular surface parameters related to dry eye disease and to evaluate their association with radiological disease severity. **Methods:** This retrospective study included 32 patients with surgery-indicated CRSwNP who underwent functional endoscopic sinus surgery (January 2021–January 2025) and 31 controls, all of whom had complete ophthalmological examinations. Ocular surface parameters, including Ocular Surface Disease Index (OSDI) scores, tear break-up time (BUT), Schirmer test values, and Oxford staining scores, were compared between the groups. Disease severity was assessed using the Sino-Nasal Outcome Test-22 (SNOT-22) and the Lund–Mackay score (LMS). Generalized estimating equations (GEE) were used for eye-level analyses. Associations between ipsilateral LMS and ocular surface parameters, as well as the effects of the degree and location of sinus involvement, were also assessed. **Results:** Higher OSDI scores (*p* = 0.001), lower BUT and Schirmer test values, and higher Oxford staining scores were observed in the CRSwNP group (all *p* < 0.05). Ipsilateral LMS was not significantly associated with ipsilateral BUT, Schirmer test values, or Oxford staining scores. No association was observed between OSDI scores and either LMS or SNOT-22 scores. No sinus region, including the osteomeatal complex, showed a significant association after correction for multiple comparisons. **Conclusions:** Surgery-indicated CRSwNP is associated with objective ocular surface dysfunction, including impaired tear film stability and reduced aqueous tear secretion. However, ocular surface findings and dry eye symptoms were not related to radiological disease burden. These findings suggest that ocular surface evaluation may be considered in patients with surgery-indicated CRSwNP, but prospective studies are needed to confirm this.

## 1. Introduction

Chronic rhinosinusitis (CRS) is defined by persistent inflammation of the nasal mucosa and paranasal sinuses lasting for 12 weeks or more. It affects approximately 5–12% of the general population, making it a significant public health concern. The primary symptom is nasal obstruction, but patients may also experience nasal discharge, postnasal drip, olfactory and gustatory dysfunction, and facial pain or pressure [1,2].

Diagnosis is based on the presence of characteristic clinical symptoms and is supported by paranasal sinus computed tomography (PNSCT) imaging or nasal endoscopy. Endoscopic findings may include nasal polyps, mucopurulent discharge—particularly from the middle meatus—and mucosal edema or obstruction. PNSCT remains the gold standard for the radiological assessment of CRS, and the Lund–Mackay scoring (LMS) system is widely used to quantify disease extent [1]. The Sinonasal Outcome Test-22 (SNOT-22) is the most commonly employed instrument for evaluating disease-specific quality of life in patients with CRS.

Based on phenotypic characteristics, CRS is classified into chronic rhinosinusitis with nasal polyps (CRSwNP) and chronic rhinosinusitis without nasal polyps (CRSsNP). In addition, CRS can be categorized into Type 2 and non-Type 2 disease according to underlying endotypic inflammatory mechanisms [1,3].

Due to the close anatomical relationship between the sinonasal cavity and the orbit, CRS may be associated with ocular manifestations over time [4]. In this context, dry eye disease (DED) has emerged as a potential comorbidity of interest. DED is a multifactorial disorder characterized by tear film instability and ocular surface inflammation, leading to symptoms such as dryness, burning, irritation, blurred vision, and ocular discomfort [5,6].

Inflammation plays a central role in the pathophysiology of both CRS and DED, suggesting a possible link between these two conditions. However, the number of studies specifically addressing the relationship between CRS—particularly CRS with surgical indications—and ocular surface dysfunction remains limited [7].

Therefore, the present study aimed to retrospectively investigate the relationship between surgically indicated CRSwNP and ocular surface parameters related to dry eye disease.

## 2. Materials and Methods

### 2.1. Study Design and Study Population

Ethical approval was obtained from the Scientific Research Ethics Committee of Karadeniz Technical University Faculty of Medicine (approval code: 2024/211; approval date: 25 June 2025). The study was conducted in accordance with the Declaration of Helsinki. In line with the policies of our tertiary university hospital, all patients provided documented institutional consent at registration or admission for medical examination and the use of their clinical information. The Ethics Committee approved the retrospective use of anonymized archival data, and permission to access the medical records was obtained from the institution before the study began. No examination or procedure was performed specifically for this study. No personally identifiable information was collected, and the data were anonymized before analysis.

Our institution is a tertiary university hospital and regional referral center for patients undergoing surgery for CRSwNP. We retrospectively reviewed the records of 573 patients who underwent surgery for nasal polyps between January 2021 and January 2025. Of these, 73 had attended the ophthalmology outpatient clinic within six months before surgery. Thirty-two patients had complete rhinological and ocular surface assessment records and met the remaining eligibility criteria; they formed the CRSwNP group. The remaining 41 patients were excluded because they did not meet the eligibility criteria or had incomplete rhinological or ocular surface assessment records. The ophthalmological visits were not arranged for this study, and the reason for attendance was not consistently recorded. CRSwNP was diagnosed from the preoperative clinical and nasal endoscopic findings and confirmed by postoperative histopathological examination.

The control group was selected retrospectively from individuals who had been examined in both our otorhinolaryngology and ophthalmology outpatient clinics between January 2021 and January 2025. It comprised 31 individuals whose ophthalmological examination took place on the same day as or within three months after their otorhinolaryngological examination and whose records included complete ocular surface and nasal endoscopic assessments. These individuals had attended the otorhinolaryngology outpatient clinic for otological complaints or neck swelling. At our institution, nasal endoscopy is routinely performed in patients with these complaints, so nasal endoscopic findings were available for all control participants. Individuals with no sinonasal symptoms or clinical findings suggestive of CRS or another sinonasal disorder were eligible for the control group. PNSCT was not performed because there was no clinical indication for imaging. Where documented, the most common reason for the ophthalmology visit was refractive error; however, because of the retrospective design, the reason for each visit could not be consistently determined.

Individuals with a history of ocular surface disease or its sequelae, previous ophthalmological surgery, active ocular surface infection, contact lens wear, allergic rhinitis, asthma, use of medications known to affect tear secretion or composition (e.g., oral contraceptives, antihistamines, parasympatholytic agents, or sympathomimetic agents), use of topical ocular medications, or systemic diseases that could affect ocular surface parameters were excluded. Patients in the CRSwNP group were not using intranasal corticosteroids at the time of the ophthalmological examination. Patients whose records did not allow these criteria to be verified were not included. The same exclusion criteria were applied to both groups.

### 2.2. Data Collection

Data collected for analysis included patients’ demographic characteristics, Lund–Mackay scores (LMSs), Sino-Nasal Outcome Test-22 (SNOT-22) scores, and postoperative histopathological findings. Ophthalmological data included bilateral tear break-up time (BUT), Oxford staining scores, Schirmer test values obtained under topical anesthesia (proparacaine hydrochloride 0.5%; Alcaine^®^; S.A. Alcon-Couvreur N.V., Puurs, Belgium), and Ocular Surface Disease Index (OSDI) questionnaire scores. All ophthalmological assessments were performed in accordance with previously established and widely accepted standardized protocols described in the literature [8].

### 2.3. Clinical and Radiological Assessment

Preoperative PNSCT scans of patients in the CRSwNP group were evaluated using the Lund–Mackay scoring (LMS) system. The PNSCT scans used for LMS had been obtained within three months before surgery. Each side of the sinonasal cavity was assessed separately and divided into six anatomical regions: the maxillary, anterior ethmoid, posterior ethmoid, sphenoid, and frontal sinuses, as well as the osteomeatal complex (OMC).

Sinus opacification was graded on a scale of 0 to 2, where 0 indicated no opacification, 1 indicated partial opacification, and 2 indicated complete opacification. The OMC was evaluated separately as either patent (0) or occluded (2). Hypoplastic or aplastic sinuses were excluded from the analysis. For each patient, ipsilateral LMS values were calculated separately for the right and left sides, yielding scores ranging from 0 (complete sinus aeration) to 12 (complete opacification of all evaluated regions on one side) [9]. Ipsilateral LMS was calculated as the sum of the six regional scores on each side, and total LMS as the sum of both sides.

### 2.4. Ophthalmological Assessment

For the CRSwNP group, bilateral BUT, Oxford staining scores, and Schirmer test results obtained under topical anesthesia were retrieved retrospectively from the medical records. Only examinations performed within six months before functional endoscopic sinus surgery were included, to exclude any possible effect of surgery on ocular surface findings. No additional ophthalmological examination was performed for this study. Findings from the right and left eyes were recorded separately and analyzed as eye-level data.

### 2.5. Questionnaires

The SNOT-22 questionnaire, a validated disease-specific patient-reported outcome measure, was used to assess quality of life in patients with CRS. Patients completed the SNOT-22 during their preoperative assessment, approximately 7–10 days before surgery. The questionnaire consists of 22 items evaluating sinonasal symptoms and their impact on daily functioning and quality of life. Each item is scored on a scale from 0 to 5, yielding a total score ranging from 0 to 110, with higher scores indicating greater disease burden and impairment in quality of life [3].

The OSDI questionnaire, a validated patient-reported outcome measure, was used to assess the severity of dry eye symptoms and their impact on vision-related functioning. The OSDI consists of 12 items scored on a 5-point Likert scale (0 = none of the time, 4 = all of the time). The total OSDI score was calculated using the standard formula (sum of scores × 25) / number of questions answered, yielding a score ranging from 0 to 100, with higher scores indicating greater symptom severity and functional impairment. Based on established cut-off values, scores of 0–12 were considered normal, 13–22 mild, 23–32 moderate, and 33–100 severe dry eye disease [10].

### 2.6. Statistical Analysis

The original analyses were performed using IBM SPSS Statistics for Windows, Version 25.0 (IBM Corp., Armonk, NY, USA), and the revised analyses were performed in Python using the statsmodels package (version 0.14.5). The normality of continuous variables was assessed using the Shapiro–Wilk test. Continuous variables were summarized as median (minimum–maximum), and categorical variables as frequencies and percentages.

Patient-level comparisons between the CRSwNP and control groups were performed using the Mann–Whitney U test for continuous variables and the chi-square test for categorical variables. Associations of OSDI with total LMS and SNOT-22 were assessed using Spearman’s rank correlation coefficient.

Given the within-subject correlation between measurements from the right and left eyes, eye-level data, including BUT, Schirmer test with topical anesthesia, and Oxford staining scores, were analyzed using generalized estimating equations (GEE), with each participant as the clustering unit and robust standard errors. Between-group comparisons were adjusted for age and sex, using an exchangeable working correlation structure. Within the CRSwNP group, the associations between ipsilateral LMS and ipsilateral ocular surface parameters were evaluated using unadjusted and age- and sex-adjusted GEE models with an exchangeable working correlation structure.

For exploratory analyses of sinus involvement, the grades of all sinus regions were entered simultaneously into GEE models, with Grade 0 (no involvement) as the reference category. These models used a working-independence correlation structure with patient-clustered robust standard errors and were not adjusted for age or sex. The overall association of each sinus region was assessed using a joint Wald test. Holm correction was applied to the between-group comparisons, the adjusted LMS analyses, and the sinus-grade contrasts. Sensitivity analyses included a small-sample corrected covariance estimator and ordinal GEE for Oxford staining scores. Beta coefficients with corresponding 95% confidence intervals and *p*-values were reported.

A two-sided *p*-value of less than 0.05 was considered statistically significant for all tests.

## 3. Results

A total of 63 participants were included in the study, comprising 32 patients in the CRSwNP group and 31 individuals in the control group. The proportion of female participants was significantly higher in the control group than in the CRSwNP group (74.2% vs. 40.6%, *p* = 0.007). The median age was 40.5 years (range, 17–61) in the CRSwNP group and 28 years (range, 18–78) in the control group, with no statistically significant difference between groups (*p* = 0.11) (Table 1).

OSDI scores were significantly higher in the CRSwNP group compared with the control group (Mann–Whitney U = 261.000, *p* = 0.001) (Table 1).

In the CRSwNP group, the median time from the eye examination to surgery was 90.5 days (range, 7–168 days), and the median time from PNSCT to surgery was 55.5 days (range, 1–91 days). The median absolute time between the eye examination and PNSCT was 54.5 days (range, 2–141 days).

Nasal obstruction was the most common presenting symptom (90.6%), followed by rhinorrhea and olfactory loss (62.5% each) and postnasal drip (56.3%). The median SNOT-22 score was 48, and the median total LMS was 13 (Table 2).

At the patient level, OSDI scores were not significantly associated with total LMS (Spearman ρ = 0.218, *p* = 0.230) or SNOT-22 scores (ρ = 0.218, *p* = 0.231).

Eye-level comparisons included 126 eyes of 63 participants. In GEE models adjusted for age and sex, BUT and Schirmer test values were significantly lower in the CRSwNP group (β = −1.65 s, 95% CI −2.89 to −0.41, *p* = 0.009; and β = −4.27 mm, 95% CI −6.38 to −2.16, *p* < 0.001, respectively), whereas Oxford staining scores were significantly higher (β = 0.61, 95% CI 0.38 to 0.83, *p* < 0.001) (Table 3). All three differences remained significant after Holm correction.

Within the CRSwNP group, the associations between ipsilateral LMS and ipsilateral ocular surface parameters were evaluated in 64 eyes of 32 patients. Ipsilateral LMS was not significantly associated with BUT (β = −0.105 s per point, 95% CI −0.231 to 0.020, *p* = 0.099), Schirmer test values (β = 0.049 mm per point, 95% CI −0.129 to 0.226, *p* = 0.592), or Oxford staining scores (β = −0.003 per point, 95% CI −0.019 to 0.013, *p* = 0.745) in age- and sex-adjusted models. The unadjusted models yielded similar results (Table 4).

The association between the degree and location of sinus involvement and ocular surface parameters was further evaluated. Descriptive values according to sinus involvement grade are presented in Table 5A. In GEE models including all six sinus regions simultaneously, the overall association between frontal sinus grade and Schirmer test values reached nominal significance (Wald χ^2^ = 6.299, df = 2, unadjusted *p* = 0.043), whereas none of the other 17 overall tests was significant (all *p* > 0.05) (Table 5B). None of the 33 individual grade comparisons with Grade 0 as the reference remained significant after Holm correction (Table S1).

For OMC involvement (Grade 2 vs. Grade 0), the estimated difference in BUT was −1.49 s (95% CI −4.21 to 1.23, *p* = 0.282). OMC involvement was also not significantly associated with Schirmer test values (β = 2.36 mm, 95% CI −1.26 to 5.99, *p* = 0.202) or Oxford staining scores (β = −0.05, 95% CI −0.84 to 0.73, *p* = 0.897) (Table 5B and Table S1).

Nominally significant differences in Schirmer test values were observed for Grade 1 frontal sinus involvement (β = 4.83 mm, *p* = 0.030) and for Grade 1 and Grade 2 maxillary sinus involvement (β = −5.36 mm, *p* = 0.034; and β = −4.83 mm, *p* = 0.048, respectively); however, none remained significant after Holm correction (all adjusted *p* ≥ 0.977) (Table S1).

Sensitivity analyses yielded consistent results. With a small-sample bias-corrected covariance estimator, the between-group differences remained significant and the adjusted LMS associations remained nonsignificant. An ordinal GEE model for Oxford staining scores supported the between-group difference (odds ratio for a higher score, 26.41; 95% CI 6.11–114.20; *p* < 0.001) but showed no significant association with ipsilateral LMS (OR 0.93; 95% CI 0.85–1.01; *p* = 0.084).

## 4. Discussion

This study demonstrated that both subjective dry eye symptoms and objective ocular surface parameters were significantly affected in patients with surgery-indicated CRSwNP compared with controls. The higher OSDI scores observed in the CRSwNP group, along with decreased BUT and Schirmer test values and increased Oxford staining scores, indicate impaired ocular surface integrity and tear film instability. The differences in BUT, Schirmer test values, and Oxford staining scores remained statistically significant after adjustment for age and sex. However, within the CRSwNP group, ipsilateral LMS was not significantly associated with any ocular surface parameter after accounting for inter-eye correlation, and no sinus region, including the OMC, showed a significant association after correction for multiple comparisons. Likewise, OSDI scores were not significantly associated with LMS or SNOT-22 scores.

Median BUT was 7 s in the CRSwNP group and 9 s in controls. Median Schirmer values were 15 and 20 mm, and Oxford staining scores were 1 and 0, respectively. Median Schirmer values in both groups remained above commonly used cut-offs for reduced aqueous tear secretion, whereas median BUT was below the conventional 10-s threshold in both groups, and corneal staining was minimal [8]. These differences therefore appear to be meaningful at the group level rather than for individual patients, and their clinical relevance should be interpreted with caution.

The clinical association between CRS and DED has also been reported in recent population-based studies. In a cohort study by Lee et al., the risk of developing DED was found to be increased among patients with surgery-indicated CRS, and persistent sinonasal inflammation was proposed as a potential trigger of the ocular surface inflammatory cycle [7]. In another study, however, Kim et al. reported structural and functional changes in the optic nerve in patients with CRS [11]. These findings suggest that CRS is not confined to the local sinonasal mucosa but may also involve adjacent orbital and ocular structures. Our study further supports this clinical association by providing objective data derived from tear function parameters.

Few studies have used objective tests to evaluate dry eye parameters in patients with CRSwNP or CRSsNP. Studies relating radiological disease burden in CRSwNP to objective ocular surface findings are also scarce. In our study, we compared the LMS of each side with the ocular surface findings of the eye on the same side.

Allergic rhinitis (AR) has been associated with DED in previous studies [12], and rhinitis symptom severity, disease duration, and nasal secretion type have been identified as risk factors for DED in patients with chronic rhinitis [13]. Mucosal inflammation may also affect tear film stability through nasolacrimal drainage [13]. Because patients with documented AR were excluded, our findings are unlikely to be explained by coexisting AR. In our study, OMC involvement was not significantly associated with BUT (*p* = 0.282), and nasolacrimal drainage was not examined.

Although ocular surface parameters differed between groups, they were not associated with radiological disease extent within the CRSwNP group. There are several possible explanations. Ocular surface changes may be more closely related to the type and activity of inflammation than to the extent of sinus involvement measured by LMS. All patients in the CRSwNP group had surgical indications, which may have limited the variation in disease severity and made an association harder to detect. The small sample size may also have limited statistical power; the confidence interval for BUT did not rule out a modest negative association with LMS. Finally, given the retrospective design and the different selection methods used for the two groups, some of the between-group differences may have resulted from selection bias or confounding rather than from CRSwNP itself.

The nominal associations between frontal and maxillary sinus involvement and Schirmer test values did not remain statistically significant after correction for multiple comparisons, and their directions were inconsistent. Given the small number of eyes in some subgroups, these findings are unlikely to represent true associations.

Inflammation plays a role in both DED and CRSwNP [1,14,15]. Type 2 inflammation in CRSwNP can affect epithelial barrier integrity [1,16], raising the possibility that shared inflammatory pathways contribute to ocular surface changes. Ocular surface homeostasis is also influenced by neural pathways. Stimulation of the nasal mucosa can affect lacrimal secretion through a trigeminal–parasympathetic reflex [17], and chronic sinonasal inflammation may alter this reflex. In addition, elevated inflammatory cytokines in the tear film and ocular surface damage have been reported in chronic inflammatory diseases such as Sjögren’s syndrome, rheumatoid arthritis, and systemic sclerosis [6,18,19,20]. Since we did not assess inflammatory biomarkers or reflex tear secretion, these mechanisms remain possible explanations for our findings.

In our study, OSDI scores were not significantly correlated with LMS or SNOT-22 scores. Discrepancies between dry eye symptoms and clinical findings have been reported previously [21,22,23]. Pain processing and corneal sensitivity may influence symptom perception and partly explain this finding [24,25], but we did not assess these mechanisms in our patients.

Ocular surface changes may occur in patients with surgery-indicated CRSwNP, but our findings do not identify a subgroup at greater risk based on radiological findings. Prospective studies are needed to determine whether routine preoperative ocular surface assessment is useful in these patients.

The strengths of this study include the use of the GEE method for the analysis of measurements obtained from both eyes, the combined evaluation of subjective and objective parameters, and the assessment of the relationship between ipsilateral radiologic disease burden and corresponding ocular surface findings.

This study has several limitations. Its retrospective design limited control over potential confounding factors and carried a risk of incomplete or inconsistent documentation. The interval between the eye examination and PNSCT was as long as 141 days in some patients. Sinonasal findings may have changed during this time. In addition, only patients with CRSwNP who had undergone ophthalmological examination before surgery and had complete ocular surface assessments in their medical records were included. The reasons for ophthalmology visits could not be consistently determined in either group. This selection may have introduced bias in both groups, and the CRSwNP group may not represent the broader population of patients with CRSwNP. The inclusion of patients with surgical indications also limits the generalizability of the findings to patients with milder disease. The exclusion of patients with asthma or allergic rhinitis, which commonly coexist with CRSwNP, further limits generalizability. Previous intranasal corticosteroid use before the ophthalmological examination could not be fully excluded. Smoking status was not consistently available and could not be included in the analysis. The relatively small sample size, particularly in some sinus subgroups, may have reduced the power to detect significant differences. No prospective sample size calculation was performed because of the retrospective design. The sinus-specific analyses were exploratory and involved multiple comparisons. The difference in sex distribution between the groups may also have affected the results, given the higher prevalence of DED among women. Adjustment for age and sex was performed in the GEE models; however, residual confounding remains possible. Inflammatory biomarkers related to ocular surface inflammation and CRS endotypes were not available. Finally, the cross-sectional analysis does not allow conclusions about causality. Further prospective studies with larger and more balanced samples are needed to confirm these findings.

## 5. Conclusions

Patients with surgery-indicated CRSwNP had lower BUT and Schirmer test values and higher Oxford staining scores than controls. These differences remained statistically significant after adjustment for age and sex. Within the CRSwNP group, ocular surface parameters were not significantly associated with ipsilateral LMS or involvement of any individual sinus region, including the OMC. OSDI scores were not significantly associated with LMS or SNOT-22 scores. Given the retrospective design and selected patient group, prospective studies are needed to confirm these findings before routine ocular surface assessment can be recommended for patients with CRSwNP.

## Figures and Tables

**Table 1:** Demographic characteristics and OSDI scores of the study groups.

Variable	Control (*n* = 31)	CRSwNP (*n* = 32)	*p*-Value
Female, *n* (%)	23 (74.2)	13 (40.6)	0.007
Age, years, median (min–max)	28 (18–78)	40.5 (17–61)	0.11
OSDI, score, median (min–max)	12.50 (0–41.60)	21.85 (0–58.30)	0.001

Values are presented as median (minimum–maximum) unless otherwise indicated. Patient-level analyses: Mann–Whitney U test for age and OSDI; chi-square test for sex.

**Table 2:** Sinonasal symptoms and disease severity parameters in patients with surgery-indicated CRSwNP.

Variable	CRSwNP Group (*n* = 32)
Nasal obstruction, *n* (%)	29 (90.6)
Rhinorrhea, *n* (%)	20 (62.5)
Facial pain/pressure, *n* (%)	11 (34.4)
Headache, *n* (%)	17 (53.1)
Postnasal drip, *n* (%)	18 (56.3)
Olfactory loss, *n* (%)	20 (62.5)
Symptom duration, months, median (min–max)	24 (4–240)
SNOT-22 score, median (min–max)	48 (7–89)
Total Lund–Mackay score, median (min–max)	13 (3–24)

Values are presented as *n* (%) or median (minimum–maximum). SNOT-22: Sino-Nasal Outcome Test-22.

**Table 3:** Comparison of ocular surface parameters between the CRSwNP and control groups.

Parameters	Control Eyes (*n* = 62)	CRSwNP Eyes (*n* = 64)	β (95% CI)	*p*-Value
BUT (s)	9 (2–13)	7 (2–13)	−1.65 (−2.89 to −0.41)	0.009
Schirmer test (mm)	20 (3–27)	15 (5–30)	−4.27 (−6.38 to −2.16)	<0.001
Oxford staining score (Grade)	0 (0–1)	1 (0–2)	0.61 (0.38 to 0.83)	<0.001

Values in the group columns are presented as median (minimum–maximum); Eye-level data were analyzed using generalized estimating equations (GEEs) with the patient as the clustering unit, an exchangeable working correlation structure, and robust standard errors. The models were adjusted for age and sex. Regression coefficients (β) represent the estimated difference in the CRSwNP group compared with the control group; for Oxford staining scores, β represents the difference in mean score. All differences remained significant after Holm correction for the three comparisons. BUT: tear break-up time; s: seconds; mm: millimeters.

**Table 4:** Association between ipsilateral Lund–Mackay score and ipsilateral ocular surface parameters in patients with surgery-indicated CRSwNP.

Parameter	Unadjusted β (95% CI)	*p*	Age/Sex Adjusted β (95% CI)	*p*
BUT, s	−0.110 (−0.232 to 0.013)	0.079	−0.105 (−0.231 to 0.020)	0.099
Schirmer test, mm	0.031 (−0.136 to 0.198)	0.717	0.049 (−0.129 to 0.226)	0.592
Oxford staining score	0.000 (−0.017 to 0.017)	0.999	−0.003 (−0.019 to 0.013)	0.745

Each model included 64 eyes of 32 patients with CRSwNP. Data were analyzed using generalized estimating equations (GEE) with the patient as the clustering unit, an exchangeable working correlation structure, and robust standard errors. β represents the estimated change in each parameter per one-point increase in ipsilateral LMS. After Holm correction across the three adjusted models, *p*-values were 0.298, 1.000, and 1.000, respectively. BUT: tear break-up time; LMS: Lund–Mackay score; s: seconds; mm: millimeters.

**Table 5:** (**A**) Ocular surface parameters according to sinus involvement grade. (**B**) Overall association of sinus involvement grade with ocular surface parameters.

**(A)**					
**Sinus**	**Grade**	**Eyes (** ***n*** **)**	**BUT (s)**	**Schirmer test (mm)**	**Oxford staining score**
Frontal	0	33	7 (2–13)	16 (5–27)	1 (0–2)
	1	8	7 (3–10)	17 (13–30)	0 (0–1)
	2	23	7 (3–13)	15 (5–27)	1 (0–2)
Anterior ethmoid	0	18	8 (2–13)	17 (5–24)	1 (0–2)
	1	15	6 (3–10)	14 (10–25)	0 (0–2)
	2	31	7 (3–13)	15 (5–30)	1 (0–2)
Posterior ethmoid	0	21	8 (2–13)	17 (5–27)	1 (0–2)
	1	25	7 (3–13)	15 (5–28)	0 (0–2)
	2	18	7 (3–10)	15 (8–30)	1 (0–2)
Maxillary	0	13	8 (2–13)	17 (5–25)	1 (0–2)
	1	22	7 (3–10)	15 (8–28)	1 (0–2)
	2	29	6 (3–13)	15 (5–30)	0 (0–2)
Sphenoid	0	31	7 (2–13)	17 (5–27)	1 (0–2)
	1	16	7 (3–10)	15 (8–27)	0 (0–1)
	2	17	7 (3–10)	14 (8–30)	0 (0–2)
OMC	0	16	8 (2–13)	17 (5–25)	0.5 (0–2)
	2	48	7 (3–13)	15 (5–30)	1 (0–2)
**(B)**					
**Sinus**		**df**	**BUT,** ***p***	**Schirmer test,** ***p***	**Oxford staining score,** ***p***
Frontal		2	0.861	0.043	0.459
Anterior ethmoid		2	0.289	0.377	0.644
Posterior ethmoid		2	0.679	0.305	0.829
Maxillary		2	0.819	0.104	0.723
Sphenoid		2	0.636	0.989	0.246
OMC		1	0.282	0.202	0.897

(A): Values are presented as median (minimum–maximum). *n* denotes the number of eyes, not independent patients. The severity of sinus involvement was graded as 0 (no opacification), 1 (partial opacification), and 2 (complete opacification). The osteomeatal complex was categorized as 0 (patent) or 2 (occluded). Statistical analyses accounting for inter-eye correlation are presented in Table 5B and Table S1. BUT: tear break-up time; OMC: osteomeatal complex; s: seconds; mm: millimeters. (B): Analyses included 64 eyes of 32 patients with CRSwNP. For each parameter, the grades of all six sinus regions were entered simultaneously as categorical predictors into a generalized estimating equations (GEE) model with a Gaussian distribution, identity link, working-independence correlation, and patient-clustered robust standard errors. Models were mutually adjusted for the other sinus regions but not for age or sex. *p*-values are from joint Wald tests of all grade coefficients for each region; they are not adjusted for multiple comparisons, and these analyses are exploratory. Individual grade comparisons are presented in Table S1. BUT: tear break-up time; df: degrees of freedom; OMC: osteomeatal complex.

## Data Availability

The data presented in this study are available from the corresponding author upon reasonable request. The data are not publicly available due to privacy restrictions and ethical considerations related to patient confidentiality.

## Supplementary Materials

The following supporting information can be downloaded at https://www.mdpi.com/article/10.3390/diagnostics16203352/s1, Table S1: Association between sinus involvement grade and ocular surface parameters.

## Abbreviations

The following abbreviations are used in this manuscript:

AR	Allergic rhinitis
BUT	Tear break-up time
CI	Confidence interval
CRS	Chronic rhinosinusitis
CRSsNP	Chronic rhinosinusitis without nasal polyps
CRSwNP	Chronic rhinosinusitis with nasal polyps
DED	Dry eye disease
GEE	Generalized estimating equations
LMS	Lund–Mackay score
OMC	Osteomeatal complex
OR	Odds ratio
OSDI	Ocular Surface Disease Index
PNSCT	Paranasal sinus computed tomography
SNOT-22	Sino-Nasal Outcome Test-22
