# Supplementary material for: Ocular Surface Findings in Patients with Surgery-Indicated Chronic Rhinosinusitis with Nasal Polyps: A Retrospective Study
Source: Diagnostics (Basel). 2026 Oct 15;16(20):3352. doi: 10.3390/diagnostics16203352 (PMC13703161; doi:10.3390/diagnostics16203352)
Supplement: Supplementary file 1 [file diagnostics-16-03352-s001.zip › diagnostics-4558139-supplementary.pdf]

Table S1: Association between sinus involvement grade and ocular surface parameters.

Sinus	Comparison (eyes)	BUT β (95% CI)	p / Holm p	Schirmer test β (95% CI)	p / Holm p	Oxford β (95% CI)	p / Holm p
Frontal	1 vs 0 (8 vs 33)	0.08 (−2.29 to 2.46)	0.945 / 1.000	4.83 (0.48 to 9.18)	0.030 / 0.977	−0.24 (−0.78 to 0.29)	0.374 / 1.000
	2 vs 0 (23 vs 33)	0.55 (−1.69 to 2.79)	0.631 / 1.000	−0.46 (−4.42 to 3.51)	0.821 / 1.000	0.01 (−0.50 to 0.51)	0.981 / 1.000
Anterior ethmoid	1 vs 0 (15 vs 18)	−1.79 (−4.01 to 0.44)	0.116 / 1.000	2.86 (−1.84 to 7.56)	0.232 / 1.000	0.11 (−0.44 to 0.65)	0.704 / 1.000
	2 vs 0 (31 vs 18)	−1.05 (−3.78 to 1.69)	0.454 / 1.000	4.04 (−1.84 to 9.93)	0.178 / 1.000	0.32 (−0.38 to 1.03)	0.369 / 1.000
Posterior ethmoid	1 vs 0 (25 vs 21)	−0.94 (−3.13 to 1.25)	0.401 / 1.000	−4.00 (−9.11 to 1.11)	0.125 / 1.000	−0.09 (−0.85 to 0.67)	0.809 / 1.000
	2 vs 0 (18 vs 21)	−1.01 (−3.65 to 1.64)	0.456 / 1.000	−4.73 (−12.08 to 2.62)	0.207 / 1.000	0.05 (−0.80 to 0.90)	0.905 / 1.000
Maxillary	1 vs 0 (22 vs 13)	0.04 (−3.37 to 3.45)	0.982 / 1.000	−5.36 (−10.32 to −0.40)	0.034 / 1.000	0.19 (−0.57 to 0.95)	0.629 / 1.000
	2 vs 0 (29 vs 13)	−0.41 (−4.43 to 3.61)	0.842 / 1.000	−4.83 (−9.62 to −0.05)	0.048 / 1.000	0.09 (−0.80 to 0.99)	0.836 / 1.000
Sphenoid	1 vs 0 (16 vs 31)	0.81 (−1.08 to 2.70)	0.402 / 1.000	0.10 (−3.79 to 3.99)	0.959 / 1.000	−0.48 (−1.06 to 0.11)	0.112 / 1.000
	2 vs 0 (17 vs 31)	0.93 (−1.05 to 2.91)	0.356 / 1.000	0.24 (−3.92 to 4.41)	0.909 / 1.000	−0.45 (−1.05 to 0.14)	0.137 / 1.000
OMC	2 vs 0 (48 vs 16)	−1.49 (−4.21 to 1.23)	0.282 / 1.000	2.36 (−1.26 to 5.99)	0.202 / 1.000	−0.05 (−0.84 to 0.73)	0.897 / 1.000

Analyses included 64 eyes of 32 patients with CRSwNP. For each parameter, the grades of all six sinus regions were entered simultaneously into a generalized estimating equations (GEE) model with working-independence correlation and patient-clustered robust standard errors to account for the correlation between the right and left eyes. Grade 0 (no opacification) was the reference category in all models. Values in parentheses in the Comparison column indicate the number of eyes in each grade. β represents the mean difference compared with Grade 0, adjusted for the other sinus regions but not for age or sex. p-values are shown as nominal p / Holm-adjusted p; Holm correction was applied across all 33 grade comparisons for the three parameters. Confidence intervals are not adjusted for multiple comparisons. All analyses are exploratory.
BUT: tear break-up time; s: seconds; mm: millimeters; OMC: osteomeatal complex.
